# Modulation of Alzheimer’s disease brain pathology in mice by gut bacterial depletion: the role of IL-17a

**DOI:** 10.1080/19490976.2024.2363014

**Published:** 2024-06-21

**Authors:** Wenlin Hao, Qinghua Luo, Inge Tomic, Wenqiang Quan, Tobias Hartmann, Michael D. Menger, Klaus Fassbender, Yang Liu

**Affiliations:** a Department of Neurology, Saarland University, Homburg/Saar, Germany; b German Institute for Dementia Prevention (DIDP), Saarland University, Homburg/Saar, Germany; c Department of Neurology, The second affiliated hospital of Nanchang University, Nanchang, China; d Department of Clinical Laboratory, Tongji Hospital, Tongji University Medical School, Shanghai, China; e Department of Experimental Neurology, Saarland University, Homburg/Saar, Germany; f Department of Experimental Surgery, Saarland University, Homburg/Saar, Germany

**Keywords:** Alzheimer’s disease, gut microbiome, microglia, amyloid pathology, and Il-17a

## Abstract

Gut bacteria regulate brain pathology of Alzheimer’s disease (AD) patients and animal models; however, the underlying mechanism remains unclear. In this study, 3-month-old APP-transgenic female mice with and without knock-out of *Il-17a* gene were treated with antibiotics-supplemented or normal drinking water for 2 months. The antibiotic treatment eradicated almost all intestinal bacteria, which led to a reduction in Il-17a-expressing CD4-positive T lymphocytes in the spleen and gut, and to a decrease in bacterial DNA in brain tissue. Depletion of gut bacteria inhibited inflammatory activation in both brain tissue and microglia, lowered cerebral Aβ levels, and promoted transcription of *Arc* gene in the brain of APP-transgenic mice, all of which effects were abolished by deficiency of Il-17a. As possible mechanisms regulating Aβ pathology, depletion of gut bacteria inhibited β-secretase activity and increased the expression of Abcb1 and Lrp1 in the brain or at the blood-brain barrier, which were also reversed by the absence of Il-17a. Interestingly, a crossbreeding experiment between APP-transgenic mice and *Il-17a* knockout mice further showed that deficiency of Il-17a had already increased Abcb1 and Lrp1 expression at the blood-brain barrier. Thus, depletion of gut bacteria attenuates inflammatory activation and amyloid pathology in APP-transgenic mice via Il-17a-involved signaling pathways. Our study contributes to a better understanding of the gut-brain axis in AD pathophysiology and highlights the therapeutic potential of Il-17a inhibition or specific depletion of gut bacteria that stimulate the development of Il-17a-expressing T cells.

## Introduction

The microbial composition in the gut undergoes alterations in both Alzheimer’s disease (AD) patients and mouse models.^1-4^ A prospective study of cognitively healthy individuals revealed that a decrease in butyrate-producing bacterial species (e.g., *Roseburia inulinivorans* and *R. faecis*) or an increase in pro-inflammatory bacteria (e.g., *Veillonella dispar* and *V. atypica*) correlates with subjective cognitive decline over a follow-up period of 2 to 4 years^5^). Transplantation of gut bacteria from AD patients to gut bacteria-depleted rats results in impaired neurogenesis and cognitive function.^6^ Gut bacteria-free Alzheimer’s precursor protein (APP)-transgenic or *App*^*NL-G-F*^ knock-in AD mice also exhibit reduced amyloid β peptide (Aβ) deposition and microgliosis in the brain.^7-11^ Consequently, gut bacteria play an important role in AD pathogenesis, though the mechanism of how gut bacteria impact brain pathology in AD remains to be investigated.

However, the specific profile of intestinal bacteria associated with AD has not yet been defined. There is often a decrease in bacterial abundance in *Firmicutes* phylum, an increase in *Proteobacteria* phylum, and both a decrease and an increase in the proportion of *Bacteroidetes* bacteria.^1,4,12,13^ At the family and genus levels, the variability of results regarding AD-specific bacterial changes is even greater across different studies. For example, a consistent decrease in bacteria of the genus *Butyricicoccus* or *Coprococcus* or an increase in the genera *Escherichia/Shigella* (three genera are not changed in the same study) in AD patients is only found in two out of twenty independent studies.^4^ *Butyricicoccus* and *Coprococcus* produce butyrate, which prevents excessive inflammation in the gut,^14,15^ while *Escherichia/Shigella* releases toxins and promotes inflammatory activation in the human body.^14^ The variability in research findings also exists in AD animals.^4^ It is a challenge to investigate the precise role of different bacterial taxa in AD pathogenesis. Germ-free or broad-spectrum antibiotic-treated mice are still often used to study the molecular mechanisms by which gut bacteria influence brain pathology in AD.

An important mechanism mediating the brain-gut axis is that gut bacteria produce short-chain free fatty acids (SCFAs, i.e., acetate, butyrate and propionate) that promote the maturation of microglia, innate immune responses and energy metabolism in a homeostatic state.^16,17^ Administration of SCFAs to germ-free or antibiotic-treated AD mice restores microglial proliferation and inflammatory activation; however, the effects on Aβ phagocytosis and Aβ accumulation in the brain are inconsistent between different studies.^16,18,19^ Whether SCFAs act directly on microglia also remains unclear. We found that SCFA receptors, G protein-coupled receptor (Gpr) 41 and Gpr43 are absent in murine microglia^20^; however, deficiency of Gpr41 and Gpr43 likely inhibits microglial maturation under physiological conditions^17^ and increases microglial density and Aβ deposits in the brain of APP-transgenic mice.^21^ Deficiency of the butyrate receptor Gpr109a, which is highly expressed in microglia,^20^ has limited effects on microglial activation.^21^ Since the SCFAs produced by intestinal bacteria activate Gpr41, Gpr43 and Gpr109a, promote anti-inflammatory properties and regulate the development of interleukin (Il)-17a or Il-10 producing T cells in the gut,^21–23^ we hypothesized that gut bacteria alter microglial activation and brain pathology through circulating T lymphocytes.

Germ-free mice generate fewer Il-17a-producing CD4(+) T help (Th17) lymphocytes but more CD4(+)CD25(+)Foxp3(+) regulatory T (Treg) cells in the gut and spinal cord, which is associated with resistance to experimental autoimmune encephalomyelitis (EAE) in mice.^24^ Depletion of intestinal bacteria by antibiotics reduces the accumulation of Il-17a-producing γδ T cells in the leptomeninges and ameliorates brain injury in a stroke mouse model.^25^ In APP-transgenic mice, reductions in cerebral Aβ deposition and microglia after gut antibiotic treatments correlate with increased levels of Foxp3+ Treg cells in blood and brain.^10^ However, transient depletion of Treg cells was shown to regulate microglia or/and infiltrated macrophages with differential effects on Aβ clearance and cognitive protection in two studies.^26,27^ Our recent study showed that deficiency of p38α-MAPK in peripheral myeloid cells decreases Th17 cells, which possibly increases microglial activation and Aβ clearance in AD mice.^28^ Therefore, we asked whether Il-17a-expressing cells mediate the effects of gut bacteria on AD pathology.

Intestinal bacteria may also release structural components into the blood and bacteria themselves may even spread in the brain, both of which can directly activate microglia. Blood concentrations of lipopolysaccharide (LPS) together with inflammatory cytokines, e.g., IL-1β and tumor necrosis factor (TNF)-α, are increased in AD patients compared to non-AD individuals with and without cognitive impairment.^29^ Components of *Porphyromonas gingivalis*, a bacterium often existing in chronic periodontitis, were found in the brains of AD patients.^30^ We have observed that bacterial receptors CD14 and Toll-like receptor (TLR)-2 are receptors for aggregated Aβ,^31–33^ implying that Aβ and bacterial components share receptors on microglia in the brain. MyD88 is an adaptor protein that is down-stream of most TLRs.^34^ Our previous studies showed that deficiency of CD14, TLR2, TLR4, or MyD88 inhibits inflammatory activation of microglia, reduces cerebral Aβ and improves cognitive function in APP-transgenic mice.^20,32,33,35,36^ Antibiotic therapy for AD patients has attracted interest.^37^ A large cohort study suggests that sporadic use of antibiotics in older adults may decrease the risk of dementia.^38^ Long-term antibiotic exposure reduces Th17 cells in the gut.^39^ The question arises as to whether inhibition of Il-17a signaling mediates the efficacy of potential antibiotic therapy in AD.

AD pathology is more likely to lead to clinical dementia in women than in men. It is also believed that the prevalence or incidence of AD is higher in women than in men. The difference is not only due to the longer life expectancy of women, but also to biological or sociocultural factors that differ between women and men.^40^ In this study, we treated female APP-transgenic mice with and without an antibiotic cocktail in the drinking water and observed that depletion of intestinal bacteria reduced inflammatory activation and Aβ load in the brain. These effects could be abolished by knockout of *Il-17a* gene.

## Materials and methods

### Animal models and cross-breeding

APP/PS1-transgenic mice (APP^tg^) over-expressing human mutated APP (KM670/671NL) and PS1 (L166P) under Thy-1 promoters^41^ were gifts from M. Jucker, Hertie Institute for Clinical Brain Research, Tübingen, Germany. *Il-17a* knockout (Il17a^−/−^) mice were kindly provided by Y. Iwakura, Tokyo University of Science, Japan.^42^ Il-17a-deficient AD mice were created by cross-breeding APP/PS1-transgenic mice and Il17a^−/−^ mice to obtain APP^tg^/Il17a^−/−^ genotype in our previous study.^28^ APP/PS1-transgenic mice were also cross-bred with Il-17a-eGFP reporter mice (Il17a^GFP/GFP^; kindly provided by R. Flavell, Yale University, USA) to get APP^tg^/Il17a^GFP/wt^ of genotype, in which eGFP is expressed under the control of endogenous *Il-17a* gene promoter.^43^ All mice used in this project were on C57BL/6 genetic background.

### Depletion of intestinal bacteria with antibiotics in drinking water

Sisters of Il-17a-deficient or wildtype APP-transgenic mice from each litter (≥2 mice per genotype) were randomly separated. Il-17a-deficient and wild-type female mice were cohoused and treated with and without vancomycin (500 mg/L), ampicillin (1 g/L), neomycin sulfate (1 g/L), streptomycin (1 g/L), and metronidazole (1 g/L) (all antibiotics were purchased from Sigma-Aldrich Chemie GmbH, Taufkirchen, Germany) in drinking water from 3 months of age for 2 months to remove gut bacteria according to a published protocol.^44^ As controls, 3 or 22-month-old C57BL/6J female mice were treated in the same way. The water with antibiotics was changed every 7 days. In order to examine the off-target effects of oral antibiotics on neuroinflammation, we injected 5-month-old APP-transgenic mice via the peritoneal cavity daily for 7 days with 1.5 mg/kg/day vancomycin, 3 mg/kg/day ampicillin, neomycin and streptomycin according to the published protocol^45^ and 30 mg/kg/day metronidazole, as it has a high oral bioavailability.^46^ Animal experiments were conducted in accordance with national rules and ARRIVE guidelines, and authorized by Landesamt für Verbraucherschutz, Saarland, Germany (registration numbers: 56/2015, 46/2017 and 34/2019) and Nanchang University, China.

### Tissue collection and isolation of blood vessels

Animals were euthanized at 5 months of age by inhalation of overdose isoflurane or by *i.p*. injection of ketamine (100 mg/kg)/xylazine (10 mg/kg). After intracardial perfusion with ice-cold PBS, the brain was removed and divided along the anterior-posterior axis. The left hemisphere was immediately fixed in 4% paraformaldehyde (Sigma-Aldrich) in PBS and embedded in paraffin for immunohistochemistry. The olfactory bulb was first removed from the right hemisphere, and the cortex and hippocampus were carefully separated from the brainstem, thalamus and striatum under microscope. A roughly 0.5-mm thick sagittal section of tissue was cut from the medial side of the tissue and homogenized in TRIzol (Thermo Fisher Scientific, Darmstadt, Germany) for RNA and DNA isolation. The remainder of the right hemisphere was snap-frozen in liquid nitrogen and stored at −80°C until biochemical analysis. The appendix together with a 0.5-cm-long segment of the neighboring colon was also collected, snap-frozen and stored at −80°C for isolation of intestinal bacteria.

To isolate brain blood vessels, the cortex and hippocampus from 5-month-old APP-transgenic mice were carefully dissected and brain vessel fragments were isolated as we did previously.^20^ Briefly, brain tissues were homogenized in HEPES-contained Hanks’ balanced salt solution (HBSS) and centrifuged at 4,400 g in HEPES-HBSS buffer supplemented with dextran from *Leuconostoc spp*. (molecular weight ~ 70,000; Sigma-Aldrich) to delete myelin. The vessel pellet was re-suspended in HEPES-HBSS buffer supplemented with 1% bovine serum albumin (Sigma-Aldrich) and filtered with 20 μm-mesh. The blood vessel fragments were collected on the top of filter and stored at −80°C for biochemical analysis.

### Intestinal bacterial collection and 16S rRNA sequencing

Bacterial DNA was extracted from intestinal bacteria (100 mg) in the frozen cecum and colon with QIAamp Fast DNA Stool Mini Kit (Qiagen, Hilden, Germany). The amount of bacteria was evaluated by quantifying 16S rRNA with SYBR Green-based real-time PCR using the universal bacterial r16S gene primers (16S-V2-101F: 5-AGYGGCGIACGGGTGAGTAA-3, and 16S-V2-361 R: 5-CYIACTGCTGCCTCCCGTAG-3) as it was conducted in a published study.^25^ The V3 -V4 region of the 16S rRNA-encoding gene was then amplified with the barcode fusion primers (338F: 5-ACTCCTACGGGAGGCAGCAG-3, and 806 R: 5-GGACTACHVGGGTWTCTAAT-3). After purification, PCR products were used for constructing libraries and sequenced on the Illumina MiSeq platform at Majorbio Co. Ltd. (Shanghai, China). The raw data was processed on Qiime2 (https://qiime2.org/.), reducing sequencing and PCR errors, and denoising to get the operational taxonomic unit (OTU) consensus sequences, which were mapped to the 16S Mothur-Silva SEED r119 database (http://www.mothur.org/). Alpha diversity including Sobs, Shannon, Ace, Chao and Simpson indexes were used for the analysis of bacterial richness and diversity in a single mouse. Principal coordinate analysis (PCoA) and analysis of similarity (ANOSIM) were used for β-diversity analysis to compare bacterial compositions on genus level between APP-transgenic mice with and without antibiotic treatments. The difference of bacterial compositions on genus level between these two groups were also compared with Wilcoxon rank sum test. All the analysis was performed using cloud-based tools with default analysis parameters (https://cloud.majorbio.com/page/tools.html).

### Quantification of bacterial DNA in the brain tissue

DNA was extracted from the brain tissue using TRIzol (Thermo Fisher Scientific) according to the protocol provided by the company. To assess the presence and extent of bacterial dissemination into the brain, real-time PCR was conducted using universal bacterial 16S rRNA gene primers (16S-V2-101F and 16S-V2-361 R), and primers targeted mouse *Gapdh* gene (sense, 5’-*ACAACTTTGGCATTGTGGAA*-3’ and antisense, 5’-*GATGCAGGGATGATGTTCTG*-3’) as an internal control.

### Positive selection of CD11b-positive and CD4-positive cells from the brain and spleen, respectively

The brain tissue (hippocampus and cortex) and spleen of 5-month-old APP-transgenic mice with and without treatments with antibiotics were prepared for single-cell suspensions using Neural Tissue Dissociation Kit (papain-based) and Spleen Dissociation Kit (mouse), respectively (Miltenyi Biotec GmbH, Bergisch Gladbach, Germany). After blocking with 50 µg/ml CD16/CD32 antibody (clone 2.4G2; BioXCell, Lebanon, USA), CD11b-positive brain cells were selected from the brain with microbeads-conjugated CD11b antibody (clone M1/70.15.11.5; Miltenyi Biotec) and CD4-positive spleen cells from the spleen with Dynabeads magnetic beads-conjugated CD4 antibody (clone L3T4; Thermo Fisher Scientific). Lysis buffer was immediately added to selected cells and total RNA was isolated using RNeasy Plus Mini Kit (Qiagen).

### Microglial Aβ phagocytosis assay

Five-month-old APP-transgenic mice received an intraperitoneal injection of 10 mg/kg methoxy-XO4 (Bio-Techne GmbH, Wiesbaden-Nordenstadt, Germany) (2 mg/ml in a 1:1 mixture of DMSO and 0.9% NaCl [pH 12] (v/v)) after treatment with and without antibiotics according to a published protocol.^47^ Methoxy-XO4 binds to β-sheet secondary structure of Aβ aggregates. Three hours later, a single cell suspension from the hippocampus and cortex was prepared using Neural Tissue Dissociation Kit (papain-based) (Miltenyi Biotec GmbH). After blocking with CD16/CD32 antibody (clone 2.4G2; BioXCell) and subsequent staining with PE-Cy5-conjugated CD11b antibody (clone M1/70; Thermo Fisher Scientific), fibrillar Aβ-containing CD11b-positive brain cells were detected by BD FACSVerse™ flow cytometry (BD Biosciences; Heidelberg, Germany).

### Flow cytometric detection of Il17a-eGFP reporter in intestinal cells

A published protocol^48^ was used to prepare single cell suspensions from both lamina propria and Peyer’s patches of the small intestine of 5-month-old APP^tg^/Il17a^GFP/wt^ mice with and without two months of antibiotic treatment. After staining with APC-conjugated rat anti-CD4 monoclonal antibody (clone: GK1.5; Thermo Fisher Scientific), eGFP-expressing CD4-positive cells were detected by BD FACSCanto™ II flow cytometry (BD Biosciences).

### Histological analysis

Serial 50-μm-thick sagittal sections were cut from the paraffin-embedded hemisphere. Four neighboring sections with 300 µm of interval were deparaffinized, labeled with rabbit anti-ionized calcium-binding adapter molecule (Iba)-1 antibody (Wako Chemicals, Neuss, Germany) and VectaStain *Elite* ABC-HRP kit (Cat.-No.: PK-6100, Vector Laboratories, Burlingame, USA), and visualized with diaminobenzidine (Sigma-Aldrich). Iba-1-positive microglia/brain macrophages were counted with Optical Fractionator in the hippocampus and cortex on a Zeiss AxioImager.Z2 microscope (Carl Zeiss Microscopy GmbH, Göttingen, Germany) equipped with a Stereo Investigator system (MBF Bioscience, Williston), as we did previously.^49^

To evaluate the cerebral Aβ level, after deparaffinization, 4 serial brain sections from each animal were stained with rabbit anti-human Aβ antibody (clone D12B2; Cell Signaling Technology Europe, Frankfurt am Main, Germany) and Cy3-conjugated goat anti-rabbit IgG (Jackson ImmunoResearch Europe Ltd. Cambridge, UK), or with methoxy-XO4 (Bio-Techne GmbH). After mounting, the whole section including hippocampus and cortex was imaged with Microlucida (MBF Bioscience) and merged. The positive staining and brain region analyzed were measured for the area with Image J tool “Analyse Particles” (https://imagej.nih.gov/ij/). The threshold for all compared samples was manually set and kept constantly. The percentage of Aβ coverage in the brain was calculated.

To determine the density of CD68-positive microglia, serial brain sections were stained with rabbit anti-CD68 antibody (clone E3O7V; Cell Signaling Technology Europe) and Cy3-conjugated goat anti-rabbit IgG (Jackson ImmunoResearch Europe Ltd.). Since the single CD68-positive cell could not be clearly recognized (see Figure 7a) and reliably counted, the percentage of CD68 coverage in the brain was calculated as for Aβ.

### Analysis of microglial morphology

For the analysis of microglial morphology, our established protocol and Fiji Image J were used.^28^ Paraffin-embedded 50-µm sagittal brain sections were used as described above. After fluorescent co-staining with Iba-1 and Aβ antibodies, total 10 Aβ plaques/mouse were randomly selected from the cortex dorsal to hippocampus and imaged under 40× objective with Z-stack scanning with 1 µm of interval. The serial images were Z-projected with maximal intensity, 8-bit grayscale transformed, Unsharp-Mask filter and despeckle-treated, and binarized to obtain a black and white image. The cells with complete nucleus and branches and without overlapping with neighboring cells were chosen for analysis. The single-pixel background noise was eliminated and the gaps along processes were filled under the view of the original image of the cell. The processed image was skeletonized and analyzed with the plugin Analyze Skeleton (2D/3D) (http://imagej.net/AnalyzeSkeleton) for the total number of primary branches, length of all branches, and the number of branch endpoints of each microglia. The whole analysis was done blinded to genotypes.

### Western blot analysis

Frozen brain tissues and blood vessel isolates were homogenized in RIPA buffer (50 mM Tris [pH 8.0], 150 mM NaCl, 0.1% SDS, 0.5% sodiumdeoxy-cholate, 1% NP-40, and 5 mM EDTA) supplemented with protease inhibitor cocktail (Roche Applied Science, Mannheim, Germany) on ice. The proteins were separated by 10% or 12% Tris-glycine SDS/PAGE. Before loading on PAGE gel, vessel preparations were sonicated. Western blots were performed using rabbit monoclonal antibody against Abcb1 (clone E1Y7S) and rabbit polyclonal antibody against Lrp1 (Cat.-No.: 64099) (both antibodies were bought from Cell Signaling Technology), as well as rabbit polyclonal antibody against claudin-5 (Cat.-No.: GTX49370; GeneTex, Hsinchu, China). The detected proteins were visualized via the Plus-ECL method (PerkinElmer, Waltham, USA). To quantify proteins of interest, rabbit monoclonal antibody against β-actin (clone 13E5) or rabbit polyclonal antibody against α-tubulin (Cat.-No.: 2144) (both antibodies were from Cell Signaling Technology) was used as a protein loading control. Densitometric analysis of band densities was performed with Image-Pro Plus software version 6.0.0.260 (Media Cybernetics, Rockville, MD, USA). For each sample, the protein level was calculated as a ratio of target protein/loading control per sample.

### Brain homogenates and ELISA assays of Aβ and Il-1β and Tnf-α

The frozen brain hemispheres were homogenized and extracted serially in Tris-buffered saline (TBS), TBS plus 1% Triton X-100 (TBS-T), guanidine buffer (5 M guanidine HCl/50 mM Tris, pH 8.0) as described in our previous study.^49^ Aβ concentrations in three separate fractions of brain homogenates were determined by Invitrogen™ Amyloid β 42 and 40 Human ELISA kits (Cat.-No.: KHB3441 and KHB3481, respectively; both from Thermo Fisher Scientific). Results were normalized on the basis of the sample’s protein concentration.

We measured concentrations of Il-1β and Tnf-α in TBS-soluble brain homogenates with ELISA kits (Cat.-No.: DY401 and DY410, respectively, from R&D systems, Minneapolis, USA). The results were also adjusted by the protein concentration in the same sample.

### Quantitative PCR for analysis of gene transcripts

Total RNA was isolated from mouse brains with TRIzol or from selected CD11b or CD4-positive cells with RNeasy Plus Mini Kit (Qiagen) and reverse-transcribed. Gene transcripts were quantified with established protocols^49,50^ and TaqMan gene expression assays of mouse *Tnf-α*, *Il-1β*, *Chemokine (C – C motif) ligand 2* (*Ccl-2*), *Il-10*, *Chitinase-like 3* (*Chi3l3*), *Mannose receptor C type 1* (*Mrc1*), *Apolipoprotein e* (*Apoe*), *Triggering receptor expressed on myeloid cells 2* (*Trem2*), *Purinergic receptor P2Y, G-protein coupled 12* (*P2ry12*), *C-X3-C motif chemokine receptor 1* (*Cx3cr1*), *Lipoprotein lipase* (*Lpl*), *C-type lectin domain family 7, member a* (*Clec7a*), *Integrin alpha X* (*Itgax*), *Il-17a*, *Interferon γ* (*Ifn-γ*), *Il-4*, *Neprilysin* and *Insulin-degrading enzyme* (*Ide*), *Activity-regulated cytoskeleton-associated protein* (*Arc*), *Glutamate ionotropic receptor NMDA type subunit 1* (*Grin1*), *Brain derived neurotrophic factor* (*Bdnf*), and *Gapdh* (Thermo Fisher Scientific).

## Statistical analysis

Data were presented as mean ± *SEM* and displayed using a scatter plot with a bar overlay in the figure, with the scatter plot representing individual data points. Means for two groups of cases were compared with two independent-samples Students *t*-test. The comparison of cerebral DNA levels of bacterial 16S rRNA gene between Il-17a-deficient and wild-type APP-transgenic mice with and without antibiotic treatment were conducted by Mann-Whitney-*U*-test, because the variables were apparently non-normally distributed. For multiple comparisons, we used one-way or two-way ANOVA followed by Bonferroni, Tukey, or Dunnett T3 *post hoc* test (dependent on the result of Levene’s test to determine the equality of variances). All statistical analyses were performed with GraphPad Prism 8 version 8.0.2. for Windows (GraphPad Software, San Diego, USA) or SPSS software for Windows (Version 26.0, IBM, Armonk, USA). Other statistical methods in microbiome analysis offered by external companies have been described above. Statistical significance was set at *p* < 0.05.

## Results

### Oral treatment with an antibiotic cocktail depletes almost all bacteria in the gut of APP-transgenic mice

**Figure 1.**
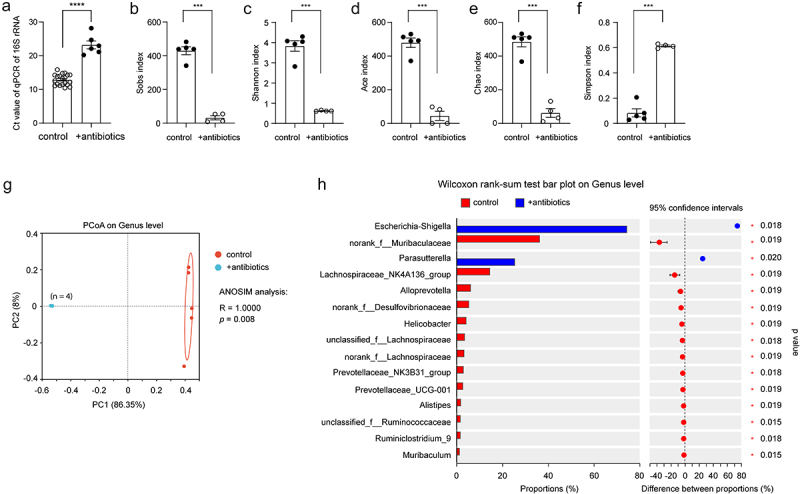
Antibiotic treatment successfully depletes the bacteria in the gut of APP-transgenic mice.

To investigate the relationship between gut and brain, we treated 3-month-old APP-transgenic female littermate mice with and without an antibiotic cocktail in drinking water for 2 months. By quantifying gut bacterial *16S rRNA* gene with real-time PCR, we found that antibiotic treatment depleted almost all bacteria in the gut, as the number of remaining bacteria was only 0.08% of that of normal drinking water control mice (Figure 1a; ΔCt value: 10.34 in real-time PCR; *t* test, *p* < 0.0001), which was consistent with previous studies that have demonstrated the depletion of gut bacteria by anaerobic and aerobic culture of gut contents.^44,51^ Not surprisingly, the richness and diversity of the remaining bacteria in the gut of antibiotics-treated AD mice were dramatically reduced, as indicated by decreased Sobs, Ace, Chao, and Shannon indices and increased Simpson index in the α-diversity analysis (Figure 1b–f; *t* test, *p* < 0.05). Similarly, the β-diversity-based PCoA analysis clearly showed the difference in intestinal bacterial architecture between APP-transgenic mice with and without antibiotic treatment (Figure 1g; ANOSIM, *R* = 1.0000, *p* = 0.008). We further observed that the remaining antibiotic-resistant bacteria belonged almost exclusively to the genera *Escherichia-Shigella* and *Parasutterella* (Figure 1h; Wilcoxon rank-sum test, *p* < 0.05).

Three-month-old APP-transgenic female mice were treated with and without antibiotics in drinking water for 2 months. Intestinal content (total 100 mg) for the isolation of bacterial DNA was harvested from the appendix and neighboring colon. Bacterial DNA was first measured for the amount with real-time PCR (a; *t* test; *n* = 20 and 6 for mice receiving normal water and antibiotic supplement), and then sequenced for the V3-V4 region of 16S rRNA-encoding gene (*n* = 5 and 4 for control and antibiotic-treated mice, respectively). Using Sobs, Shannon, Ace, Chao and Simpson’s indices, α-diversity analysis shows that treatment with an antibiotic cocktail significantly reduces bacterial richness and diversity within each mouse (b–f; *t* test). Principal coordinate analysis (PCoA) was used for β-diversity analysis of bacterial composition at the genus level in APP-transgenic mice with and without antibiotic treatment (g; Each symbol represents the gut bacteria of an individual mouse). As expected, the structure of gut bacterial community of antibiotics-treated APP-transgenic mice differed significantly from those of APP-transgenic littermates with normal drinking water (g; ANOSIM analysis between +antibiotics and control mice). Bar plots depict abundance (% of total) of the indicated genera. Wilcoxon rank-sum tests show that antibiotic treatment increases the relative abundance of bacteria in the genera *Escherichia-Shigella* and *Parasutterella* (h). ***: *p* < 0.001 and ****: *p* < 0.0001.

### Depletion of gut bacteria reduces Il-17a-expressing CD4-positive lymphocytes in APP-transgenic mice

**Figure 2.**
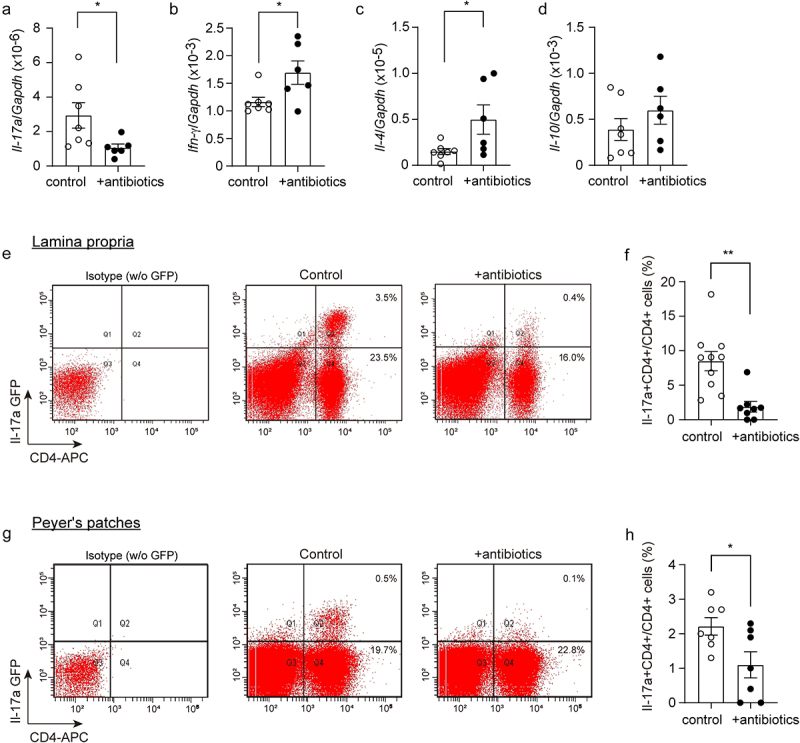
Depletion of gut bacteria reduces Il-17a-expressing CD4-positive lymphocytes.

Our recent study showed that Il-17a-expressing CD4-positive lymphocytes increase in the gut and spleen of APP-transgenic mice.^28^ We therefore selected CD4-positive cells with a magnetic beads-conjugated antibody from the spleen of antibiotics-treated APP-transgenic mice and quantified the transcripts of characteristic genes for Th1, Th2, Th17 and Treg lymphocytes. As shown in Figure 2a–d, depletion of gut bacteria significantly decreased the transcription of *Il- 17a*, but increased the transcription of *Ifn-γ* and *Il-4* (*t* test, *p* < 0.05). We further treated 3-month-old APP-transgenic mice expressing Il-17a-eGFP reporter^43^ with and without antibiotics, and observed that depletion of gut bacteria for 2 months significantly reduced eGFP-expressing CD4+ lymphocytes in both lamina propria and Peyer’s patches of the gut compared to control AD mice with normal drinking water (Figure 2e–h; *t* test, *p* < 0.05). Therefore, depletion of intestinal bacteria leads to a significant reduction in Il-17a-expressing CD4-positive T lymphocytes in AD mice. We also found some Il-17a-eGFP-expressing CD4-negative cells in both lamina propria and Peyer’s patches (see upper-left quadrant in Figure 2e and g); however, these cells could not form a clearly distinguishable population in both the antibiotics-treated and non-treated AD mice, which made it difficult to reliably quantify cells. Thus, our results do not exclude the possibility that depletion of gut bacteria also regulates Il-17a expression in other immune cells, such as lymphoid-tissue inducer-like cells, natural killer cells, and Paneth cells.^52^ In our APP-transgenic animal models, expression of GFP was not detected in γδ T lymphocytes in the gut.^28^

Three-month-old APP-transgenic female mice, expressing or not Il-17a-eGFP reporter, were treated with and without antibiotics in drinking water for 2 months. CD4-positive splenocytes were selected and detected for transcription of T lymphocyte marker genes. The transcription of *Il-17a* gene was significantly down-regulated by antibiotic treatment, while the transcription of *Ifn-γ* and *Il-4* genes was up-regulated in CD4+ splenocytes compared to APP-transgenic control mice with normal drinking water (a–c; *t* test, *n* = 6 - 7 per group). The transcription of *Il-10* gene in spleen cells was not changed by antibiotic treatment (d; *t* test, *n* = 6 - 7 per group). In addition, single cell suspensions were prepared from both lamina propria and Peyer’s patches of 5-month-old Il-17a-eGFP-expressing APP-transgenic mice and analyzed by flow cytometry after fluorescent staining of CD4 (e and g). Intestinal cells prepared from APP-transgenic mice without expressing Il-17a-eGFP reporter were stained with APC-conjugated rat IgG2b as an isotype control (Isotype w/o GFP). The expression of IL-17a-associated eGFP was decreased in CD4+ lymphocytes in the intestine of APP-transgenic mice by antibiotic treatments (f and h; *t* test, *n* = 7 - 10 per group). *p < 0.05; **p < 0.01.

In additional experiments, we performed an immunohistochemical analysis of GFP in the brain of APP-transgenic mice and of EAE mice that were established in our previous study,^53^ both of which expressed Il-17a-eGFP. We could not detect GFP-expressing cells in the brain parenchyma of AD mice, but clearly saw GFP-positive cells surrounding blood vessels in the EAE model (Supplementary Fig. S1, A). Similarly, we did not detect *Il-17a* gene transcripts in the brain tissue from APP-transgenic mice within 40 cycles of real-time PCR (data not shown). These results suggest that gut bacterial depletion alters brain pathology by regulating Il-17a-expressing cells outside the brain.

### Depletion of gut bacteria reduces bacterial DNA in the brain of APP-transgenic mice

**Figure 3.**
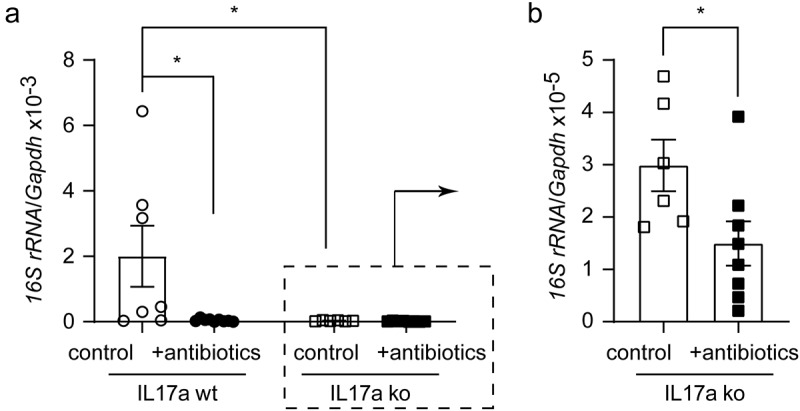
Depletion of gut bacteria reduces bacterial DNA in the brains of both Il-17a-deficient and wild-type APP-transgenic mice.

Intestinal bacteria may release structural components into the blood that circulate to the brain, or the bacteria spread directly to the brain.^30,54^ We then asked whether depletion of gut bacteria alters the potential presence of bacterial components in the brain. Since we observed that depletion of gut bacteria reduced the number of Il-17a-expressing lymphocytes, we asked whether Il-17a affected the amount of bacterial substance in the brain. We treated Il-17a-deficient and wild-type APP-transgenic female mice with antibiotics as described above. Both groups of mice after antibiotic treatment showed enlargement of the ceca and dark-colored cecal contents as shown in published studies^17^ (data not shown). Interestingly, antibiotic treatment significantly decreased the DNA level of the bacterial *16S rRNA* gene in the brains of both Il-17a-deficient and wild-type APP-transgenic mice (Figure 3a and b; Mann-Whitney-*U*-Test, *p* < 0.05). The DNA level of *16S rRNA* gene was significantly lower in Il-17a-deficient than in Il-17a-wild-type APP-transgenic mice without antibiotic treatment (Figure 3a and b; *16S rRNA*/*Gapdh*: 2.99 ± 0.49 × 10^−5^ vs. 2.00 ± 0.93 × 10^−3^; Mann-Whitney-*U*-Test, *U* = 4, *p* = 0.015). After antibiotic treatment, the DNA level of the *16S rRNA* gene did not differ between Il-17a-deficient and wild-type AD mice (*16S rRNA*/*Gapdh*: 1.49 ± 0.42 × 10^−5^ [Il-17a-deficient] vs. 4.32 ± 1.32 × 10^−5^ [Il-17a-wild-type]); Mann-Whitney-*U*-Test, *U* = 19, *p* = 0.102). Thus, Il-17a deficiency potentially blocked the translocation of bacterial components from the intestine to the brain.

Three-month-old Il-17a-deficient (ko) and wild-type (wt) APP-transgenic female mice received drinking water with and without supplement of antibiotics for 2 months. The hippocampus and cortex were collected and homogenized in Trizol for DNA isolation. The amount of bacterial DNA was evaluated by real-time PCR using mouse *Gapdh* gene as an internal control. Depletion of gut bacteria significantly decreased bacterial DNA in both Il-17a-deficient and wild-type APP-transgenic mice; interestingly, deficiency of Il-17a also reduced bacterial DNA in the brain compared with Il-17a wild-type AD mice (a and b; Mann-Whitney-*U*-Test, *n* = 8 - 9 per group for Il-17a-wildtype mice and 6 - 8 per group for Il-17a-deficient mice). *, *p*< 0.05.

### Depletion of intestinal bacteria inhibits proinflammatory activation in the brain of APP-transgenic mice, but not in Il-17a-deficient AD mice

**Figure 4.**
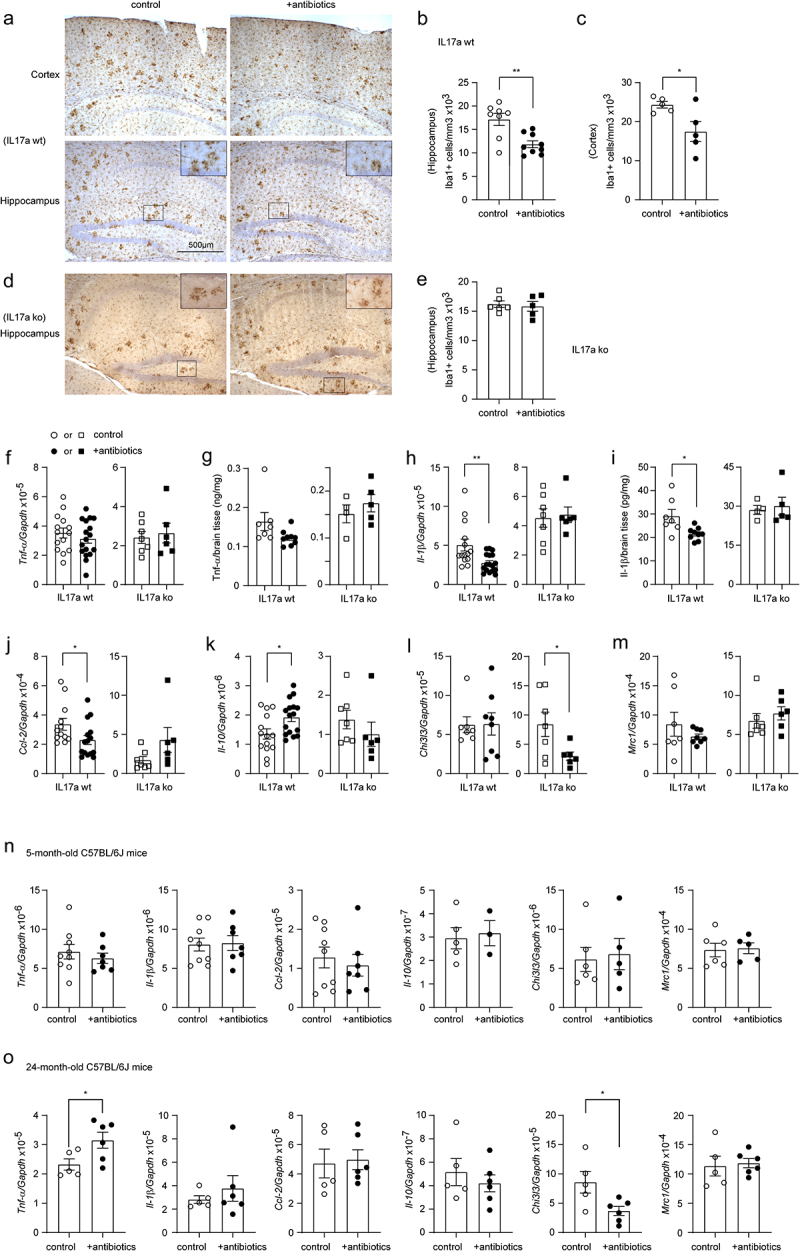
Depletion of gut bacteria reduces inflammatory activation in the brain of Il-17a-wildtype, but not Il17a-deficient APP-transgenic mice.

Depletion of intestinal bacteria has been shown to suppress inflammation in the brain of APP-transgenic mice.^8,11^ We did observe that intestinal antibiotic treatment for 2 months significantly reduced the number of Iba1-positive microglia in both the hippocampus and cortex of female APP-transgenic mice (Hippocampus: from 17.13 ± 1.29 × 10^3^ cells/mm^3^ to 11.85 ± 0.73 × 10^3^ cells/mm^3^, and Cortex: from 24.31 ± 0.84 × 10^3^ cells/mm^3^ to 17.46 ± 2.55 × 10^3^ cells/mm^3^; Figure 4, a – c; *t* test, *p* < 0.05). Interestingly, in Il-17a-deficient APP-transgenic mice, depletion of gut bacteria did not alter the number of Iba-1-positive cells in the hippocampus (Figure 4, d and e; 16.21 ± 0.57 × 10^3^ cells/mm^3^ vs. 15.85 ± 0.83 × 10^3^ cells/mm^3^; *t* test, *p* > 0.05). We measured transcripts of proinflammatory genes (*Tnf-α*, *Il-1β*, and *Ccl-2*) and anti-inflammatory genes (*Il-10*, *Chi3l3*, and *Mrc1*) in brains of APP-transgenic mice. As shown in Figure 4, f, h and j – m, depletion of intestinal bacteria down-regulated the transcription of *Il-1β* and *Ccl-2*, but up-regulated *Il-10* transcription in Il-17a-wildtype APP-transgenic mice (*t* test, *p* < 0.05). Intestinal bacterial depletion did not change the transcription of *Tnf-α*, *Chi3l3* and *Mrc-1* in APP-transgenic mice (Figure 4, f, l and m; *t* test, *p* > 0.05). In Il-17a-deficient AD mice, depletion of intestinal bacteria did not modulate the transcription of *Tnf-α*, *Il-1β*, *Ccl-2*, *Il-10*, and *Mrc-1* (Figure 4, f, h, j, k and m; *t* test, *p* > 0.05), except decreasing the transcription of *Chi3l3* (Figure 4, l; *t* test, *p* < 0.05). The results on antibiotics-induced transcriptional regulations of *Tnf-α* and *Il-1β* genes were confirmed by ELISA measurements of their protein levels, which showed that depletion of gut bacteria significantly decreased the Il-1β protein concentration (Figure 4, i; *t* test, *p* < 0.05) and tended to reduce the amount of Tnf-α protein (Figure 4, g; *t* test, *p* = 0.075) in brains of Il-17a-wildtype but not Il-17a-deficient mice.

Three-month-old APP-transgenic female mice with (ko) and without (wt) knockout of *Il-17a* gene, and 3 or 22-month-old C57BL/6J female mice were treated with and without antibiotics in drinking water for 2 months. Thereafter, brain tissues were sectioned and microglia were counted with stereological method, Optical Fractionator, after immunohistochemical staining of Iba1 (in brown color) (a–e), or homogenized for RNA isolation (f, h, and j–m) and measurement of inflammatory gene transcripts with real-time PCR, as well as for ELISA assays of Tnf-α and Il-1β concentrations (g and i). Depletion of gut bacteria significantly decreased Iba1-positive microglia in both the hippocampus and cortex of Il-17a-wildtype, but not in Il17a-deficient APP-transgenic mice (b, c and e; *t* test, *n* = 5 - 9 per group). Similarly, depletion of gut bacteria significantly reduced the transcripts of *Il-1β* and *Ccl-2*, and increased *Il-10* transcript in the brain of Il-17a-wildtype, but not in Il17a-deficient APP-transgenic mice (h, j, and k; *t* test, *n* = 6 - 17 per group). Transcriptional regulation in AD mice deleted of gut bacteria was confirmed by the reduction in protein levels of Il-1β but not Tnf-α in brain homogenate compared to APP mice receiving normal water (g and i; *t* test, *n* = 4 - 9 per group). However, depletion of gut bacteria significantly reduced the transcript of *Chi3l3* in the brain of Il17a-deficient APP-transgenic mice (l; *t* test, *n* = 6 - 7 per group). Moreover, depletion of gut bacteria did not change the transcription of various inflammatory genes (*Tnf-α*, *Il-1β*, *Ccl-2*, *Il-10*, *Chi3l3*, and *Mrc1*) in in the brain of 5-month-old C57BL/6J female mice (n; *t* test, *n* = 3 - 9 per group), however, significantly increased *Tnf-α* transcription and decreased *Chi3l3* transcription in 24-month-old C57BL/6J female mice (O; *t* test, *n* = 5 - 6 per group). *: *p* < 0.05; **: *p* < 0.01.

In order to investigate whether the intestinal bacterial depletion-induced inflammatory inhibition was specific for mice with AD pathology, we treated 3 and 22-month-old female C57BL/6J mice with and without antibiotics for 2 months. Depletion of intestinal bacteria did not change transcription of all genes tested in 5-month-old C57BL/6J mice (*Tnf-α*, *Il-1β*, *Ccl-2*, *Il-10*, *Chi3l3*, and *Mrc1*; Figure 4, n; *t* test, *p* > 0.05). In 24-month-old C57BL/6J mice, depletion of intestinal bacteria increased *Tnf-α* transcription and decreased *Chi3l3* transcription (Figure 4, o; *t* test, *p* < 0.05). These experiments also suggested that it was intestinal bacterial depletion instead of antibiotics themselves modifying the inflammatory activation in the brain of APP-transgenic mice.

In order to examine the off-target effects of oral treatment of antibiotics, we injected 5-month-old APP-transgenic mice daily for 7 days with the antibiotic cocktail (1.5 mg/kg/day vancomycin, 3 mg/kg/day ampicillin, neomycin and streptomycin) according to the published protocol.^45^ We injected metronidazole at the maximal dose of 30 mg/kg/day, because it has a high oral bioavailability.^46^ Intraperitoneal injection of the antibiotic cocktail did not change the transcription of proinflammatory genes, *Tnf-α*, *Il-1β*, and *Ccl-2* (Supplementary Fig. S2, A – C; *t* test, *p* > 0.05); however, significantly decreased the transcription of anti-inflammatory genes, *Il-10*, and *Mrc1* (Supplementary Fig. S2, D and F; *t* test, *p* < 0.05), and tended to inhibit *Chi3l3* gene transcription (Supplementary Fig. S2, E; *t* test, *p* = 0.055). The pattern of transcriptional modification in the brain by intraperitoneal injection of antibiotic cocktail is obviously different from that in oral antibiotics-treated mice; therefore, the effects of oral antibiotic treatment were due to the depletion of bacteria in the gut and not the antibiotics themselves.

### Depletion of intestinal bacteria inhibits microglial activation in the brain of APP-transgenic mice, which is abolished by knockout of Il-17a gene

**Figure 5.**
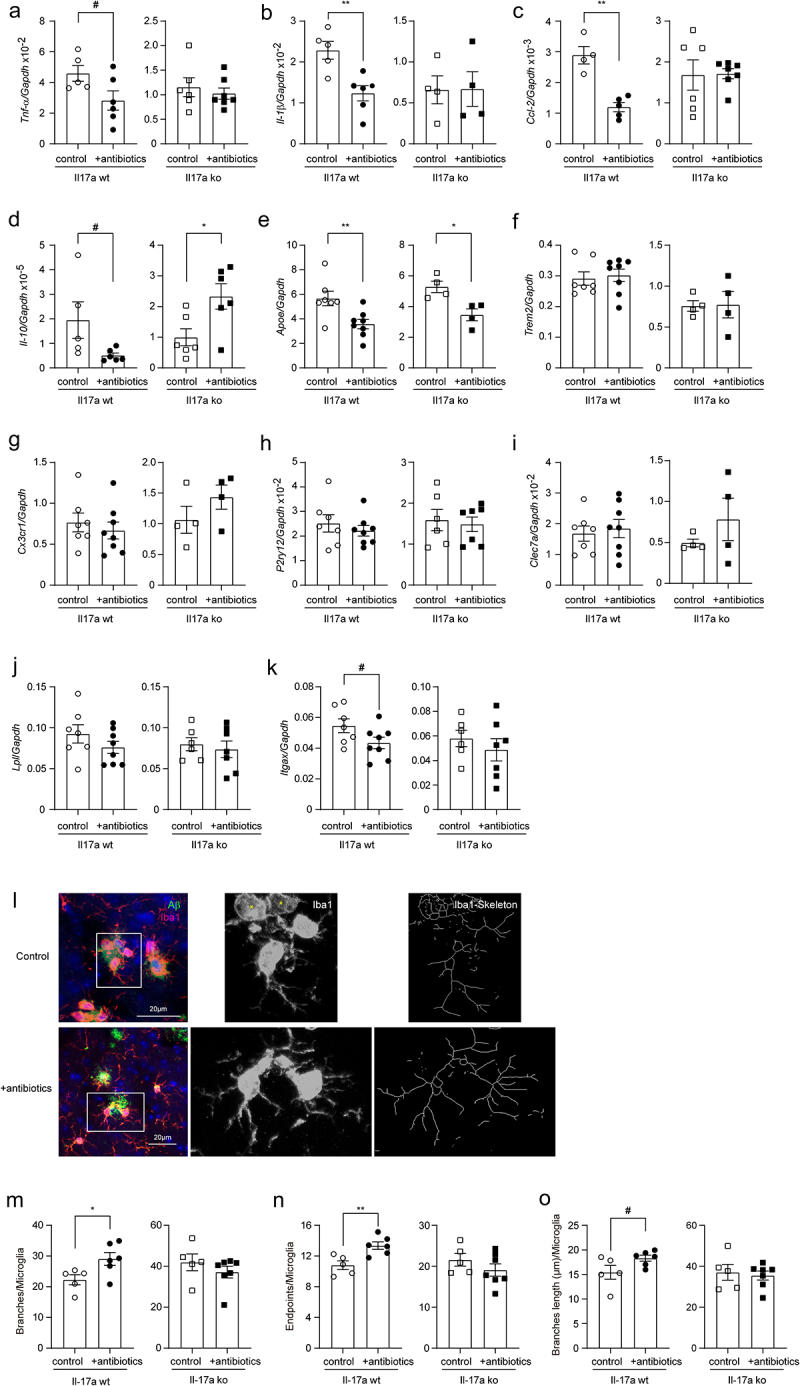
Depletion of gut bacteria inhibits inflammatory activation in microglia in the brain of Il-17a-wildtype, but not Il17a-deficient APP-transgenic mice.

To understand the mechanism, through which intestinal antibiotic treatment modified neuroinflammation in AD mice, we selected CD11b^+^ brain cells from 5-month-old female APP-transgenic mice with and without 2-month treatments of antibiotics and detected transcripts of disease-associated microglia (DAM) signature genes.^55^ Depletion of intestinal bacteria significantly reduced transcription of *Il-1β* and *Ccl-2* genes (Figure 5, b and c; *t* test, *p* < 0.05), and tended to decrease the transcription of *Tnf-α*, *Il-10* and *Itgax* genes (Figure 5, a, d and k; *t* test, 0.05 < *p* < 0.10) in cells derived from Il-17a-wildtype APP-transgenic mice, but not in cells from Il-17a-deficient AD mice. Depletion of intestinal bacteria even increased transcription of *Il-10* gene in Il-17a-deficient APP-transgenic mice (Figure 5, d; *t* test, *p* < 0.05). Gut bacterial depletion down-regulated transcription of *Apoe* gene in both Il17a-deficient and wild-type APP-transgenic mice (Figure 5, e; *t* test, *p* < 0.05). Antibiotic treatment did not significantly change the transcription of other genes (*Trem2*, *Cx3cr1*, *P2ry12*, *Clec7a*, and *Lpl*) tested in both Il-17a-deficient and wild-type mice (Figure 5, f – k; *t* test, *p* > 0.05).

Three-month-old APP-transgenic female mice with (ko) and without (wt) knockout of *Il-17a* gene were treated with and without antibiotics in drinking water for 2 months. CD11b-positive brain cells were selected and quantified for the transcription of DAM marker genes. Depletion of gut significantly decreased the transcription of *Il-1β*, and *Ccl-2* genes, and tended to down-regulate the transcription of *Tnf-α*, *Il-10*, and *Itgax* genes in the brain of Il-17a-wildtype, but not in Il17a-deficient APP-transgenic mice (a–d, and k; *t* test, *n* = 4 – 7 per group). Depletion of gut bacteria significantly reduced transcription of *Apoe* gene, but not *Trem2*, *Cx3cr1*, *P2ry12*, *Clec7a*, and *Lpl* genes in both Il-17a-wildtype and deficient APP-transgenic mice (e–j; *t* test, *n* = 4 - 8 per group). In the analysis of microglial morphology after immunofluorescent staining of Iba1 (l), depletion of gut bacteria increased the number of branches and endpoints of the processes of microglia in Il-17a-wildtype, but not in Il17a-deficient APP-transgenic mice (m – o; *t* test, *n* = 5-7 per group). The Iba1-positive cells marked with “*” without showing clear processes were excluded for analysis of microglial morphology (l). *: *p* < 0.05; **: *p* < 0.01; #: 0.05 < *p* < 0.10.

In further experiments, we labeled microglia with Iba-1 antibodies and analyzed the morphology of microglia in the vicinity of Aβ deposits as we had done previously.^28^ Depletion of intestinal bacteria increased the total number and end points of branching microglial processes (Figure 5, l – n; *t* test, *p* < 0.05), and tended to increase branch length (Figure 5, o; *t* test, *p* = 0.078) in 5-month-old APP-transgenic mice, consistent with previous observations.^16,19^ However, all changes in microglial morphology caused by gut bacteria depletion were absent in Il-17a-deficient AD mice (Figure 5, m – o; *t* test, *p* > 0.05).

As we observed bacterial DNA in the brain, we further investigated whether Il-17a deficiency alters the response of microglia to bacterial components. We found that 5-month-old Il-17a-deficient and wild-type APP-transgenic mice without antibiotic treatment did not differ in the transcription of *Myd88*, *Cd14*, *Tlr1*, *Tlr2*, *Tlr4* and *Tlr9* (Supplementary Fig. S1, B), suggesting that knockout of *Il-17a* gene does not change microglial reactivity to bacteria. Surprisingly, our additional experiments and a previous study showed that the lack of Il-17a did not reduce the transcription of inflammatory genes, *Tnf-α*, *Il-1β*, *Ccl-2* and *Il-10* in microglia (Supplementary Fig. S1, C) and even activate microglia.^28^

### Depletion of intestinal bacteria reduces cerebral Aβ in Il-17a-wildtype but not in Il-17a-deficient APP-transgenic mice

**Figure 6.**
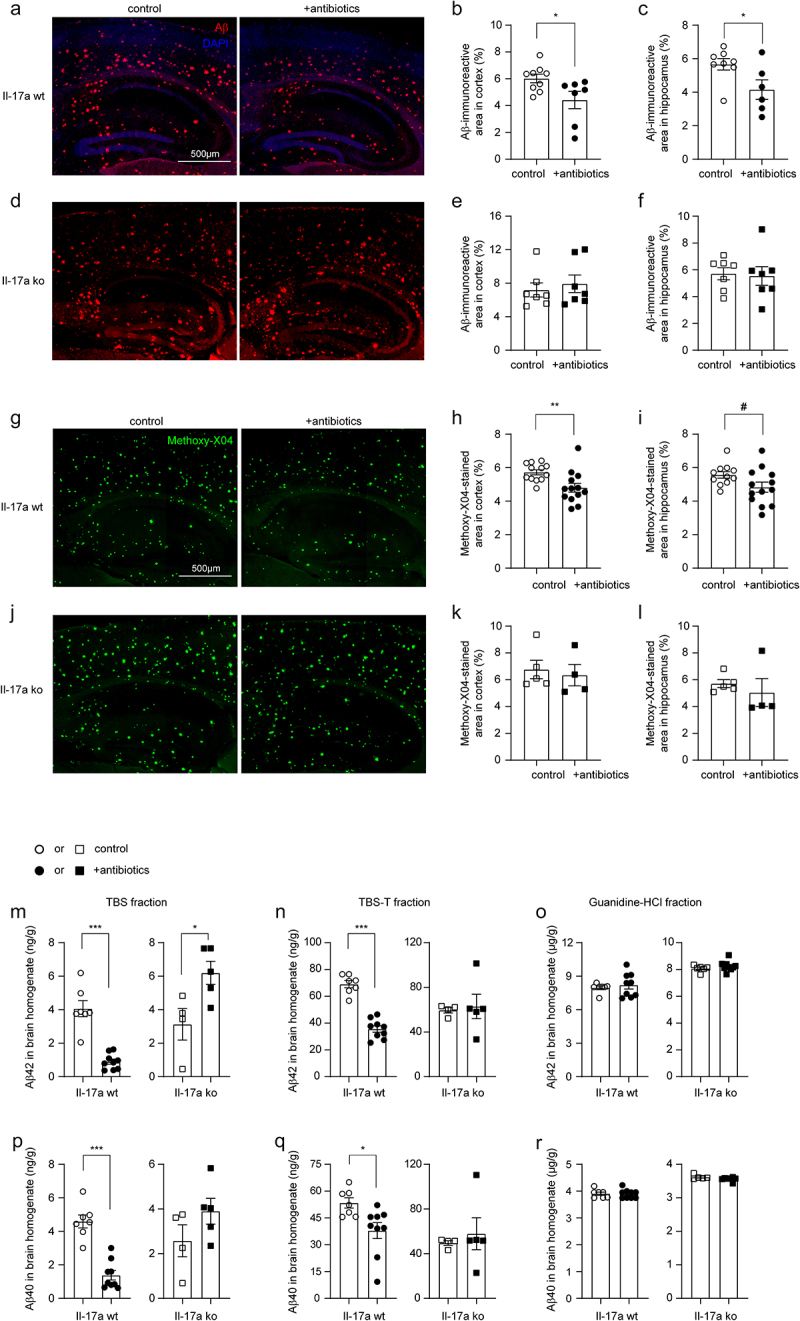
Depletion of gut bacteria reduces Aβ in the brain of Il-17a-wildtype, but not Il17a-deficient APP-transgenic mice.

Extracellular Aβ plaques are a pathological hallmark of AD. Depletion of gut bacteria was reported to attenuate Aβ deposits in APP-transgenic mice.^7,11^ We treated 3-month-old female APP-transgenic mice with an antibiotic cocktail for 2 months. As shown in Figure 6, a–c, 2-month treatments with antibiotics significantly reduced immunoreactive Aβ density from 6.02% ± 0.32% to 4.42% ± 0.64% in the cortex and from 5.67% ± 0.34% to 4.16% ± 0.58% in the hippocampus (*t* test, *p* < 0.05) of Il-17a-wildtype AD mice. In Il-17a-deficient APP-transgenic mice, treatment with antibiotics changed the density of Aβ in neither hippocampus nor cortex (Figure 6, d – f; *t* test, *p* > 0.05). The brain section was also stained with methoxy-XO4 that typically binds to the β sheet structure of Aβ aggregates. Similarly, treatments with antibiotics significantly reduced Aβ deposits in the cortex (Figure 6, g and h; *t* test, *p* < 0.05) and tended to decrease Aβ in the hippocampus (Figure 6, g and i; *t* test, *p* = 0.060) of Il-17a-wildtype APP-transgenic mice, but did not change density of methoxy-XO4-stained Aβ deposits in Il-17a-deficient AD mice (Figure 6, j – l; *t* test, *p* > 0.05).

Three-month-old APP-transgenic female mice with (ko) and without (wt) knockout of *Il-17a* gene were treated with and without antibiotics in drinking water for 2 months. Brain tissues were sectioned and stained with immunofluoresce-conjugated Aβ antibodies (a and d) and methoxy-XO4, a fluorescent dye for Aβ aggregates (g and j). Depletion of gut bacteria decreased Aβ deposits in both the hippocampus and cortex after staining of Aβ either with antibody or dye (b, c, h and i; *t* test, *n* = 6 - 13 per group). However, in the Il17a-deficient AD mice, depletion of gut bacteria did not change the cerebral Aβ loads (e, f, k and l; *t* test, *n* = 4-7 per group). Brain tissues were also serially homogenized and extracted in TBS, TBS plus 1% Triton-100 (TBS-T) and guanidine-HCl, and then measured for Aβ40 and Aβ42 with ELISA (m–r). Depletion of gut bacteria decreased the concentrations of both Aβ40 and Aβ42 in TBS- and TBS-T-soluble brain fractions, but not in guanidine-HCl-soluble fraction of Il-17a-wildtype APP-transgenic mice (m–r; *t* test, *n* = 7-9 per group). Depletion of gut bacteria did not change the concentrations of Aβ40 and Aβ42 in various fractions of brain homogenates of Il-17a-deficient AD mice (n–r; *t* test, *n* = 4 - 5 per group), except that it significantly increased the concentration of Aβ42 in TBS-soluble fraction (m). *: *p* < 0.05; **: *p* < 0.01; ***: *p* < 0.001; #: 0.05 < *p* < 0.10.

We went on measuring Aβ in the brain homogenate with ELISA. In Il-17a wild-type APP-transgenic mice, antibiotic treatment significantly decreased the levels of both Aβ42 and Aβ40 in TBS- and TBS-T fractions (Figure 6, m, n, p and q; *t* test, *p* < 0.05), but not in guanidine-soluble fraction (Figure 6, o and r; *t* test, *p* > 0.05). TBS-, TBS-T-, and guanidine-soluble brain homogenates are enriched with monomeric, oligomeric and high-molecular-weight aggregated Aβ, respectively.^33^ In Il-17a-deficient APP-transgenic mice, treatment with antibiotics did not reduce Aβ42 and Aβ40 in all three fractions of brain homogenates (Figure 6, m – r). On the contrary, antibiotic treatment even increased the concentration of Aβ42 in TBS-soluble fraction of brain homogenate (Figure 6, m; *t* test, *p* < 0.05).

### Depletion of intestinal bacteria does not increase microglial Aβ phagocytosis and extracellular Aβ degradation, but potentially reduces Aβ production

**Figure 7.**
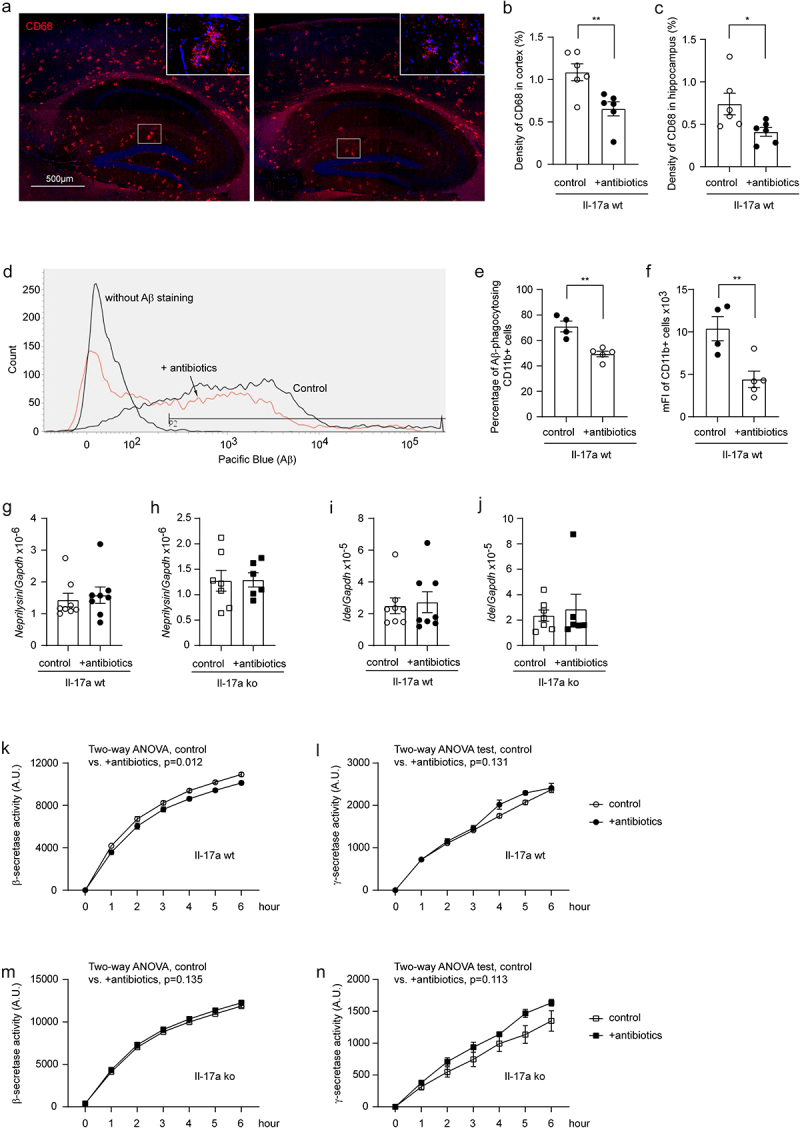
Depletion of gut bacteria reduces β-secretase activity in the brain of Il-17a-wildtype, but not Il17a-deficient APP-transgenic mice.

In following experiments, we asked how depletion of intestinal bacteria attenuated the amyloid pathology in AD mice. First, we stained brain tissue with an antibody targeting the lysosomal protein CD68, a suggested marker of phagocytosis. As shown in Figure 7, a–c, depletion of gut bacteria significantly decreased the density of CD68 immunofluorescence (*t* test, *p* < 0.05). We then conducted a flow cytometric analysis of brain cells after Aβ staining with methoxy-X04, in which depletion of intestinal bacteria remarkedly decreased both percentage of Aβ-positive CD11b+ brain cells and the mean fluorescence intensity (mFI) of CD11b+ cell population in APP-transgenic mice (Figure 7, d – f; *t* test, *p* < 0.05), apparently indicating that microglial internalization of Aβ did not contribute to cerebral Aβ clearance in gut bacteria-depleted AD mice.

Three-month-old Il-17a-deficient (ko) and wild-type (wt) APP-transgenic female mice were treated with and without antibiotics in drinking water for 2 months. The brain tissue was stained with Cy3-conjugated CD68 antibody (a). Antibiotic treatment significantly reduces the density of CD68-immunofluorescence in both cortex and hippocampus of APP-transgenic mice compared to AD mice drinking normal water (b and c; *t* test, *n* = 6 per group). APP-transgenic mice were also injected (*i.p*.) with methoxy-XO4 for the detection of Aβ-associated fluorescence in CD11b-positive brain cells by flow cytometry (d). Depletion of gut bacteria significantly decreased both the percentage of fluorescent cells among CD11b-positive brain cells, and the mean fluorescence intensity (mFI) of CD11b-positive cell population (e and f; *t* test, *n* = 4 - 5 per group). Brain tissues were further collected from AD mice for quantification of *Neprilysin* and *Ide* gene transcripts (g–j), and for β- and γ-secretase assays (k – n). Depletion of gut bacteria did not change the transcription of *Neprilysin* and *Ide* genes (g–j; *t* test, *n* = 6-8 per group); however, significantly inhibited the activity of β-secretase in the brain of Il-17a-wildtype but not Il17a-deficient APP-transgenic mice (k and m; two-way ANOVA, *n* = 6-7 per group for Il-17a-wildtype mice, and *n* = 4-5 per group for Il-17a knockout mice). Depletion of gut bacteria did not change γ-secretase activity in AD mice (l and n; two-way ANOVA, *n* = 6-7 per group for Il-17a-wildtype mice, and *n* = 4-5 per group for Il-17a knockout mice). *: *p* < 0.05; **: *p* < 0.01.

In the same APP-transgenic mouse strain as we used in this study, another group has reported that the absence of gut bacteria increased protein levels of Aβ-degrading enzymes neprilysin and Ide.^8^ However, our study showed that depletion of intestinal bacteria altered neither *Neprilysin* nor *Ide* gene transcription in the brains of Il-17a-deficient and wildtype APP-transgenic mice (Figure 7, g–j; *t* test, *p* > 0.05), which suggested that extracellular degradation of Aβ was not the mechanism mediating Aβ reduction in our AD mice.

Our previous study has shown that inhibition of neuroinflammation inhibits β- and γ-secretase activity in the brain of AD mice.^20^ We found that depletion of intestinal bacteria slightly but significantly decreased β-, but not γ-secretase activity (Figure 7, k and l; two-way ANOVA, antibiotic treatment vs. control, *p* < 0.05), as observed in a published study.^18^ It was not surprising that the same inhibitory effects of antibiotic treatment on β- and γ-secretase activity could not be seen in Il-17a-deficient APP-transgenic mice (Figure 7, m and n; two-way ANOVA, antibiotic treatment vs. control, *p* > 0.05), as the antibiotic treatment did not change the inflammatory activation in the brain of Il-17a-deficient AD mice (see Figure 4).

### Depletion of intestinal bacteria potentially increases Aβ efflux through blood-brain-barrier in APP-transgenic mice, which is driven by Il-17a inhibition

**Figure 8.**
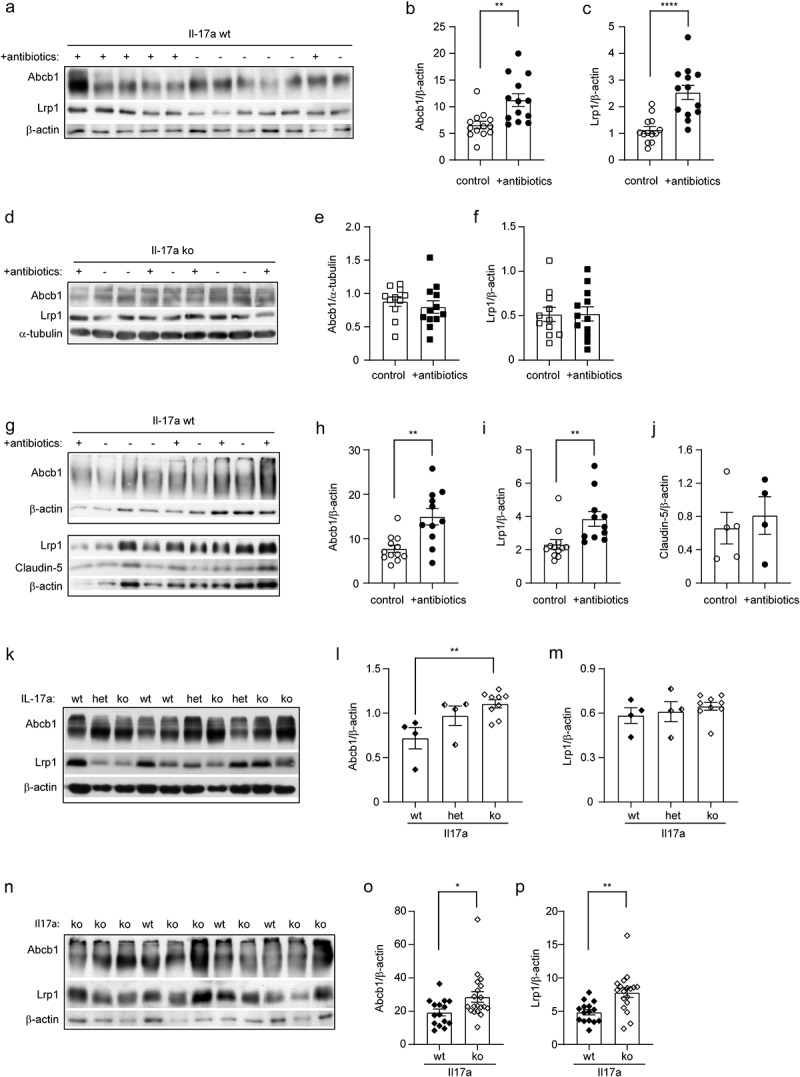
Depletion of gut bacteria increases Abcb1 and Lrp1 expression in the blood-brain-barrier of Il-17a-wildtype, but not Il17a-deficient APP-transgenic mice.

The transportation of Aβ from brain parenchyma to peripheral circulation is an efficient pathway for the cerebral Aβ clearance.^56^ LRP1 and ABCB1 are a couple of key transporters at the BBB that are responsible for Aβ efflux.^57,58^ We found that the protein levels of Abcb1 and Lrp1 were significantly increased in 5-month-old APP-transgenic mice, which had been treated with antibiotics for 2 months (Figure 8, a–c; *t* test, *p* < 0.05). Remarkably, the up-regulation of both Lrp1 and Abcb1 in the brain homogenate by depletion of intestinal bacteria was again abolished by knockout of Il-17a gene (Figure 8, d–f; *t* test, *p* > 0.05).

Three-month-old Il-17a-deficient (ko) and wild-type (wt) APP-transgenic female mice were treated with and without antibiotics in drinking water for 2 months. Brain homogenates were quantified for protein levels of Abcb1 and Lrp1 with Western blot (a and d). Depletion of gut bacteria significantly increased expression of Abcb1 and Lrp1 in brains of Il-17a-wildtype (b and c), but not in Il-17a-deficient (e and f) APP-transgenic mice (*t* test, *n* = 13, and 11 - 12 per group for Il-17a wt and ko mice, respectively). Cerebral vessels were also isolated from Il17a-wildtype AD mice and detected for Abcb1 and Lrp1 at the BBB (g). Depletion of gut bacteria significantly increased the expression of Abcb1 and Lrp1 in blood vessels of APP-transgenic mice (h and I; *t* test, *n* = 11-12 per group); however, it did not change the protein level of claudin-5 (j; *t* test, *n* = 4-5 per group). In further experiments, Abcb1 and Lrp1 were detected with quantitative Western blot in the brain homogenates from 5-month-old non-APP-transgenic female mice with different expression of Il-17a (k; wild-type [wt], heterozygote [het] and knockout [ko]), and in the isolated blood vessels from 5-month-old Il17a-wt and ko APP-transgenic female mice (n). Deficiency of Il-17a significantly increased Abcb1 but not Lrp1 in the non-APP-transgenic mouse brain in a gene dose-dependent manner (l and m; one-way ANOVA followed by Bonferroni *post-hoc* test, *n* = 4-9 per group), and increased protein levels of both Abcb1 and Lrp1 in the blood vessels of APP-transgenic mice (o and p; *t* test, *n* = 15 - 19 per group). *: *p* < 0.05; **: *p* < 0.01; ****: *p* < 0.0001.

We also isolated blood vessels from 5-month-old APP-transgenic mice with and without treatments with antibiotics. We validated the results from the entire brain homogenate that treatments with antibiotics strongly elevated protein levels of Abcb1 and Lrp1 at the BBB (Figure 8, g–i; *t* test, *p* < 0.05). Notably, the protein level of claudin-5 in cerebral blood vessels did not differ between APP-transgenic mice with and without antibiotic treatment (Figure 8, g and j; *t* test, *p* > 0.05). Similarly, we did not detect any mouse IgG in brain homogenates of antibiotics-treated AD mice with Western blot (data not shown), which suggests that depletion of intestinal bacteria increased expression of Abcb1 and Lrp1 at the BBB, but did not significantly impair the BBB.

In further experiments, we detected Lrp1 and Abcb1 in the brain homogenates from 5-month-old APP-non-transgenic female mice with different expression of Il-17a. We observed that deficiency of Il-17a significantly increased the protein level of Abcb1, but not Lrp1, in a gene dose-dependent way (Figure 8, k–m; one-way ANOVA, *p* < 0.05). Similarly, we isolated blood vessels from Il-17a-deficient and wild-type APP-transgenic mice and observed that deficiency of Il-17a significantly increased both Lrp1 and Abcb1 in the tissue lysate of blood vessels (Figure 8, n–p; *t* test, *p* < 0.05), suggesting that depletion of intestinal bacteria potentially increases Lrp1/Abcb1-mediated Aβ efflux through inhibiting Il-17a signaling.

### Depletion of intestinal bacteria potentially promotes synaptic plasticity in APP-transgenic mice, which is abolished by deficiency of Il-17a

**Figure 9.**
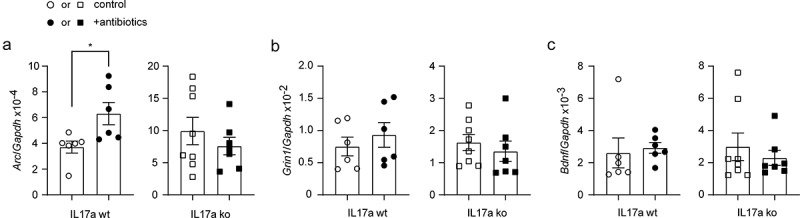
Depletion of gut bacteria increases Arc transcription in the brain of Il-17a-wildtype, but not Il17a-deficient APP-transgenic mice.

After finding that gut bacterial depletion attenuated inflammatory activation and amyloid pathology in the brains of APP-transgenic mice, we performed a preliminary analysis of the neuroprotective effect of antibiotic treatment. Our previous study has shown that upregulated transcription of the immediate early gene *Arc* and the gene *Grin1*, which encodes subunit 1 of the NMDA-type ionotropic glutamate receptor, is associated with the improvement of cognitive function in APP-transgenic mice.^35^ BDNF plays an important role in the maintenance of synaptic plasticity in learning and memory,^59^ the transcript of which is influenced by gut bacteria.^60^ Therefore, we measured the transcripts of *Arc*, *Grin1* and *Bdnf* genes in brains of 5-month-old APP-transgenic mice with and without 2-month treatment of antibiotics. As shown in Figure 9, a–c, treatment with the antibiotic cocktail significantly increased the transcription of *Arc*, but not *Grin1* and *Bdnf* genes in Il-17a-wildtype APP mice (*t* test, *p* < 0.05). Again, knockout of *Il-17a* gene abolished the neuroprotective effect of antibiotic treatment (Figure 9, a–c; *t* test, *p* > 0.05). However, the neuroprotective effects of antibiotic treatment should be further investigated by other methods, such as behavioral tests and electrophysiological approaches, in older (e.g., 9-month-old) APP-transgenic mice as we did previously.^20,28^

Three-month-old Il-17a-deficient (ko) and wild-type (wt) APP-transgenic female mice were treated with and without antibiotics in drinking water for 2 months. Thereafter, brain tissues were homogenized and measured for transcripts of *Arc*, *Grin1* and *Bdnf* genes. Antibiotic treatment significantly increased the transcription of *Arc* gene in Il-17a-wildtype, but not in Il17a-deficient APP-transgenic mice (a; *t* test, *n* = 6 and 7 - 8 per group for Il-17a wt and ko mice, respectively). Antibiotic treatment did not change the transcription of *Grin1* and *Bdnf* genes (b and c; *t* test, *n* = 6 and 7 - 8 per group for Il-17a wt and ko mice, respectively). *: *p* < 0.05.

## Discussion

Gut bacteria contribute to AD development,^61^ but how gut bacteria regulate brain pathology remains unclear. By using young female APP-transgenic mice with rapidly developing Aβ pathology, we found that depletion of gut bacteria decreased microglial inflammatory activation and Aβ levels, particularly soluble Aβ, and might promote synaptic plasticity in the brain. These effects were abolished by knockout of *Il-17a* gene. The attenuation of cerebral Aβ pathology by gut bacterial depletion may be attributed to the inhibition of β-secretase activity and the upregulation of Aβ efflux-related Lrp1 and Abcb1 expression in the brain, which was also abrogated by Il-17a deficiency.

Bacterial phenotypes are potentially transferred between contacting mice.^86^ We co-housed 4–6 Il-17a-deficient and wild-type female AD mice in the same cage for treatments with and without antibiotics, which reduced the variability caused by different housing conditions. Depletion of gut bacteria in APP-transgenic mice inhibited proinflammatory activation in the brain tissue as evidenced by reduction of microglial number, down-regulation of *Il-1β* and *Ccl-2* transcription and up-regulation of *Il-10* transcription, consistent with published studies.^8,10,18^ In individual microglia, depletion of gut bacteria decreased transcripts of both pro- (e.g., *Tnf-α*, *Il-1β*, *Ccl-2*) and anti-inflammatory (*Il-10*) genes, which is also in agreement with the published finding that the absence of gut bacteria leads to global defects in microglial activation.^17^ The opposing regulation of *Il-10* transcription in microglia and brain tissue suggests that brain cells other than microglia, such as astrocytes, may also respond to gut bacteria^62^ and produce Il-10 in the brain.

We noted that intestinal bacterial depletion in 5-month-old C57BL/6J mice did not have the same effect on the inflammatory modulation as in AD mice, and even promoted inflammatory activation (e.g., transcriptional up-regulation of *Tnf-α*, and down-regulation of *Chi3l3*) in the brain of 24-month-old C57BL/6J mice. In fact, the mechanism leading to inflammatory activation in the brain, and particularly in microglia, is not the same in AD and healthy aging. For example, inflammation in aging microglia may be due to a reduced ability to clear endogenous metabolites, whereas microglia in AD mice respond efficiently to exogenous Aβ. A transcriptomic study^63^ identified 85 aging-associated genes in female C57BL/6J mice, of which only 21 genes overlapped with DAM genes that were identified in APP-transgenic mouse model,^55^ with the exception of the DAM-characteristic genes *Trem2* and *Apoe*. Thus, it stands to reason that a significant inflammatory modulation caused by gut bacterial depletion depends on the preactivated status and pattern of inflammatory brain cells (e.g., microglia).

Depletion of gut bacteria resulted in a significant reduction of Il-17a-expressing CD4-positive cells in the gut and spleen as well as bacterial DNA in brain tissue, suggesting that gut bacteria regulate brain pathology in AD mice via at least two pathways: 1) regulating peripheral Il-17a-expressing T lymphocytes and 2) directly modulating cells in the brain with bacterial components (not necessary living cells). Very interestingly, we also observed that deficiency of Il-17a reduces bacterial DNA in the brain of APP-transgenic mice. Due to our limited research resource, we have not yet succeeded in the microbiome analysis of brain tissues. The recent study detected *Streptococcus*, *Clostridium*, *Roseburia* and *Tyzzerella* in the brain of APP/PS1 mice.^54^ The bacteria from *Cutibacterium acnes* were found in the brain of AD patient and linked to AD pathogenesis.^64^ How the bacteria or their structural components enter the brain is unclear. It has been reported that deficiency of Il-17a alters the composition of gut bacteria, i.e., increases the abundance of *Barnesiella* genus bacteria, and hinders the development of EAE.^65^ *Barnesiella* bacteria are the main source of hypoxanthine in the gut. Intestinal epithelial cells utilize hypoxanthine for energy balance and nucleotide biosynthesis.^66^ Thus, Il-17a deficiency may favor intestinal barrier function and lead to the reduction of bacterial DNA in the brain of APP-transgenic mice. Inhibition of Il-17a signaling, which can be achieved by administering Il-17a inhibitors or by depleting specific bacterial taxa in the gut that stimulate the development of Il-17a-producing T cells, therefore has the potential to avoid bacterial overload in the brain and prevent AD progression.

Exactly how the depletion of bacteria in the gut and subsequent Il-17a inhibition, in particular the reduction of Th17 cells, regulates microglial activity in our antibiotic-treated AD mice remains unclear. Il-17a inhibition itself does not appear to inhibit microglial activation. We have recently observed that knockout of *Il-17a* gene reduces microglial branches in APP-transgenic mice,^28^ suggesting activation of microglial inflammatory response.^67^ Our current study shows that Il-17a deficiency did not reduce the transcription of *Tnf-α*, *Il-1β*, *Ccl-2* and *Il-10* genes in isolated microglia (Supplementary Fig. S1, C). The inhibition of inflammation in both brain tissue and microglia in our antibiotic-treated AD mice may be due to the depletion of bacteria other than Il-17a-promoting taxa in the gut. In Il-17a-deficient APP-transgenic mice, antibiotic treatment did not inhibit inflammatory activation in brain tissue and microglia, for which there are perhaps several reasons. Deficiency of Il-17a reduced soluble Aβ (discussed later) and bacterial DNA, minimizing the inflammatory priming of microglia as in 5-month-old C57BL/6J mice, limiting the capacity to further inhibit inflammation by depleting gut bacteria. However, we believe that there are other unknown mechanisms by which Il-17a inhibition maintains and even enhances microglial activity in the brain, which we will investigate in the future studies. To answer this question, it is helpful to identify the Il-17a signaling-promoting bacteria or their metabolites in the gut of AD mice. Unfortunately, the available relevant results for today are very limited or controversial. For example, the bacterial product valeric acid was reported to increase the concentrations of Il-17, Il-1β, and Il-6 in the blood and brain of mice with experimental stroke.^68^ However, treatment with valeric acid was also shown to reduce the concentrations of Tnf-α and Il-6 in the blood of mice after irradiation.^69^ In addition, activation of the valeric acid receptor Gpr43 inhibited neuroinflammation and improved cognitive function in APP-transgenic mice.^21^

It should be noted that microglial activation is a double-edged sword in AD pathogenesis: on the one side, it releases cytotoxic cytokines and reactive oxygen species, leading to neuronal damage; on the other side, it clears neurotoxic Aβ via microglial internalization, thereby safeguarding neurons.^70^ In our recent study, p38α-MAPK deficiency in myeloid cells or Il-17a deficiency promotes microglial activation and Aβ clearance, which improves cognitive performance in APP-transgenic mice.^28^ We and other groups have also shown that stimulation of TLR4 and TLR9 by injection of a low dose of LPS or synthetic oligodeoxynucleotides containing unmethylated CpG dinucleotides, similar to those found in bacterial DNA, induces mild and long-term microglial activation that facilitates the clearance of Aβ and hyperphosphorylated tau protein in human APP or tau -transgenic mice,^71–74^ and in squirrel monkeys.^75^ Our present study has shown that general depletion of gut bacteria reduces microglial phagocytosis of Aβ in APP-transgenic mice, which is consistent with previous observations that gut bacteria are essential for microglial maturation, activation and Aβ phagocytosis.^16–19^ We would expect that specific depletion of Il-17a-promoting gut bacteria could maintain microglial activity and help clear the pathogenic Aβ molecules from the AD brain.

When studying the gut and brain, how gut bacteria modulate cerebral Aβ load is always an important question to answer. Our present study showed that microglia are not responsible for cerebral Aβ reduction in APP-transgenic mice after depletion of gut bacteria. Interestingly, we observed that depletion of gut bacteria increased the expression of Lrp1 and Abcb1 in the BBB of APP-transgenic mice, which was reversed by knockout of *Il-17a* gene. Knocking out *Il-17a* gene completely mimicked the effects of gut bacteria depletion on the expression of Lrp1 and Abcb1. Lrp1 and Abcb1 are two important transporters responsible for Aβ efflux across the BBB.^57,58^ Depletion or inhibition of endothelial Lrp1 and Abcb1 leads to Aβ accumulation in the AD mouse brain.^76,77^ Since transportation of Aβ from brain parenchyma to peripheral plasma might contribute 25% of total clearance of cerebral Aβ,^56^ Aβ efflux represents a major pathway mediating the reduction of Aβ in the brain of AD mice following depletion of gut bacteria. It has been reported that the absence of ApoE retains less Aβ in the brain parenchyma and increases soluble Aβ in the interstitial fluid, possibly promoting the diffusion of soluble Aβ from the parenchyma into the perivascular space,^78^ which favors Aβ efflux. Notably, depletion of gut bacteria decreased *Apoe* gene transcription in microglia, as observed in our study and by other scientists,^18^ which may indicate the cooperation between microglia and BBB in the clearance of cerebral Aβ.

Aβ is produced by β- (BACE1) and γ-secretases after serial digestions of APP. The published studies by our group and others have shown that the activity or protein level of β- and γ-secretases is regulated by inflammatory activation in the brain.^20,79,80^ Not surprisingly, depletion of gut bacteria inhibited β-secretase activity in correlation with inflammatory inhibition in our APP-transgenic mice, which is consistent with the observation in germ-free AD mice.^18^ We did not detect an increase in the expression of neprilysin and Ide in our APP-transgenic mice after antibiotic treatment, suggesting that extracellular degradation of Aβ is not the key mechanism for Aβ clearance after gut bacterial depletion. This result differs from the observation in a study on germ-free AD mice.^8^

Antibiotic therapy for AD patients has gained interest.^37^ In several studies, germ-free APP-transgenic mice show better cognitive function and lower cerebral Aβ levels than specific pathogen-free (SPF) AD mice.^18,81^ Our study supports antibiotic therapy, as gut bacterial depletion reduced Aβ burden in AD mice and, in particular, increased Aβ efflux-associated Abcb1 and Lrp1 across the BBB. Depletion of gut bacteria also up-regulated the transcription of *Arc* gene in the brain, which is associated with synaptic plasticity and cognitive improvement in APP-transgenic mice.^35,82^ However, as discussed above, the general depletion of gut bacteria inhibits the activation of microglia, which decreases the efficiency of Aβ clearance. In addition, gut bacteria have also been shown to promote the development of cognitive performance.^83^ Adult germ-free mice transplanted with fecal microbiota from 2–3-month-old mice perform better cognitively than mice transplanted with fecal microbiota from 18–20-month-old mice.^84^ Treatment with broad-spectrum antibiotics can deplete as well the taxa of gut bacteria that favor learning and memory. Our study also indicated that bacteria belonging to the genera *Escherichia-Shigella* and *Parasutterella* were more resistant to antibiotic treatment. The possible expansion of these bacteria, especially *Escherichia-Shigella* bacteria, after long-term antibiotic treatment could increase inflammatory activation in the brain and accelerate the progression of AD. The combination of our results and the literature indicates that it is important to manipulate a specific bacterial taxon, e.g. by reducing the bacteria that stimulate immune cells to produce Il-17a. Currently, antibiotic therapy targeting specific bacterial taxa is still a major challenge for AD patients.

A limitation of our study is that the direct anti-inflammatory effects of antibiotics orally used to deplete gut bacteria could not be excluded. Although oral vancomycin, neomycin, streptomycin and ampicillin have low systemic absorption,^60^ the oral bioavailability of metronidazole is high (98.9%).^46^ Metronidazole must not be omitted from the antibiotic cocktail as it decimates anaerobic bacteria in the gut. Metronidazole has the potential to suppress the activity of both innate and acquired immune systems.^85^ We observed that intraperitoneal injection of the antibiotic cocktail inhibited inflammatory gene transcription in the brain; however, the affected genes were mainly limited to anti-inflammatory *Il-10* and *Mrc1* genes (see Supplementary Figure S2), with a pattern different from the inflammatory regulation caused by oral treatment of antibiotics. We also observed that oral antibiotic treatment decreased *Il-17a* transcription but increased transcription of *Ifn-γ* and *Il-4* genes in CD4-positive spleen cells in APP-transgenic mice and promoted inflammatory activation in the brains of 24-month-old C57BL/6J mice. Indeed, oral antibiotic (amoxicillin/clavulanate) treatment of mice with antibiotic-sensitive and -resistant bacteria in the gut has shown that depletion of sensitive gut bacteria inhibits the development of Il-17a-expressing γδ T cells.^25^ Therefore, we have reason to believe that the inhibition of neuroinflammation and Th17 development in our AD animal models was not due to the direct effects of antibiotics, but was a consequence of the depletion of gut bacteria. The potential inhibitory effect of antibiotics on immune cells is a common pitfall in antibiotic-treated mice in many gut and brain studies.^7,17,19^

In summary, our results are consistent with published observations that depletion of gut bacteria attenuates microglial inflammatory activation and Aβ pathology in the brain of APP-transgenic mice; however, we further elucidated the possible mechanisms mediating the gut-brain axis in AD pathogenesis: 1) depletion of gut bacteria reduces peripherally circulating Il-17a-expressing T lymphocytes and translocation of bacterial components from the gut to the brain. Il-17a deficiency inhibits this bacterial translocation; 2) inhibition of Il-17a possibly maintains and even enhances microglial activity; 3) inhibition of Il-17a upregulates the expression of Lrp1 and Abcb1 in the BBB, possibly promoting Aβ efflux and Aβ clearance in the brain; and 4) depletion of gut bacteria has the potential to improve neuronal plasticity. Our study supports antibiotic therapy for AD patients; however, precise therapy targeting specific bacterial taxa is still a huge challenge. New methods and future work are needed to characterize the AD-specific microbiome profile in the gut. Particular attention should be paid to how Il-17a-associated intestinal bacteria can be manipulated.

## Supplementary Material

Supplementary Figures 220424.docx

## Data Availability

Our study has not generated new datasets. All data generated or analyzed during this study are included in this published article and its supplementary information files.
