# Supplementary material for: Modulation of Alzheimer’s disease brain pathology in mice by gut bacterial depletion: the role of IL-17a
Source: Gut Microbes. 2024 Jun 21;16(1):2363014. doi: 10.1080/19490976.2024.2363014 (PMC11195493; doi:10.1080/19490976.2024.2363014)
Supplement: Supplementary Figures 220424.docx [file KGMI_A_2363014_SM9694.docx]

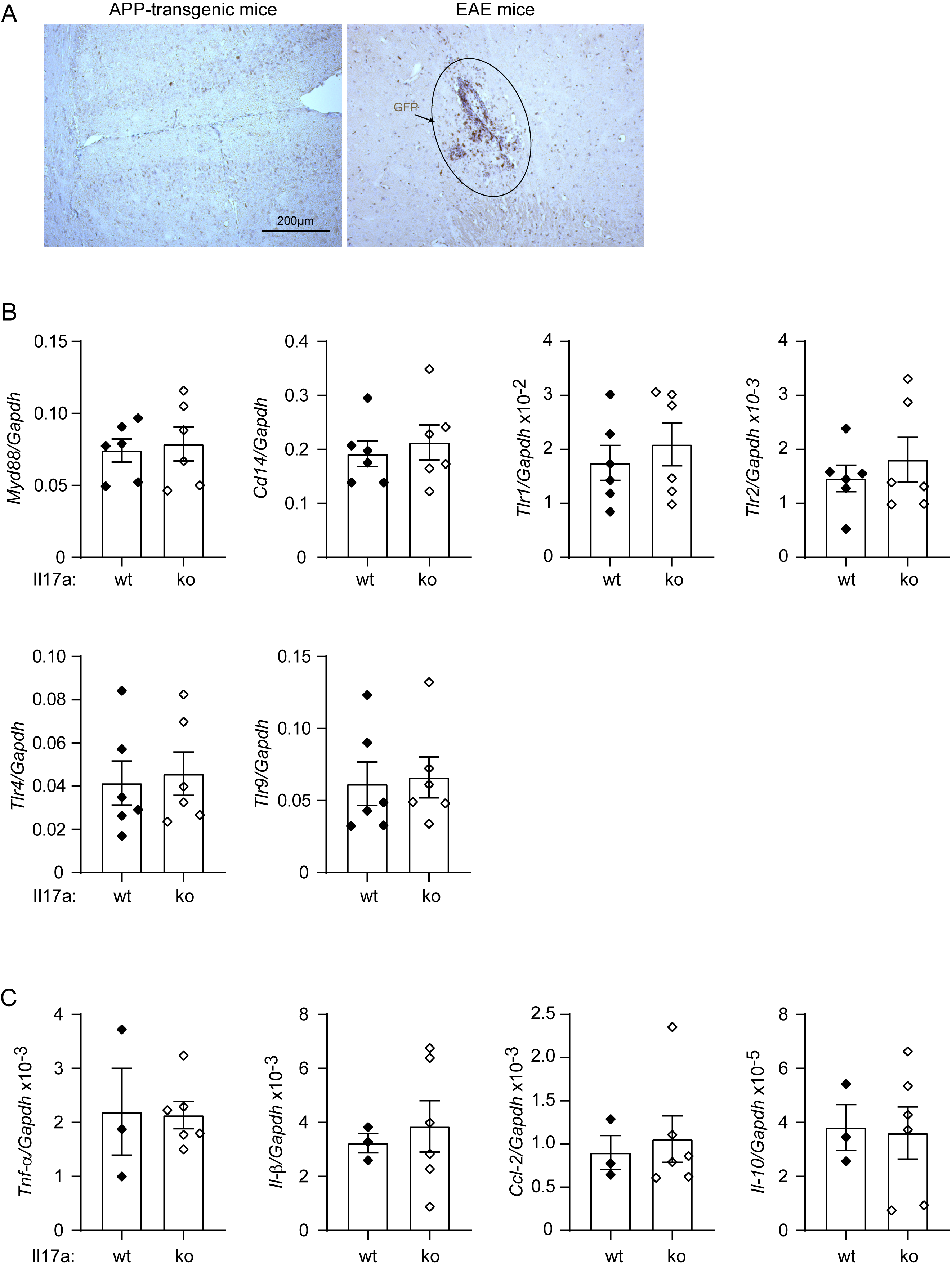


**Supplementary Figure 1, Il-17a-eGFP is not expressed in the brain and deficiency of Il-17a does not change the transcription of innate immune receptors and inflammatory genes.**

**A,** Brain sections from 5-month-old APP-transgenic mice and EAE mice both expressing Il-17a-eGFP were detected for GFP protein with immunohistochemistry. Rabbit anti-GFP antibody (Cat.-No.: 600-901-215; Rockland Immunochemicals) was used. The staining was visualized with HRP/3,3’-diaminobenzidine in brown (DAB; Sigma).

**B and C,** CD11b-positive cells were selected from single cell preparations from brains of 5-month-old *Il-17a* gene-knocked out (ko) and wild-type (wt) APP-transgenic mice with magnetic beads-conjugated antibody (clone M1/70.15.11.5; Miltenyi Biotec) and detected for transcripts of various innate immune receptor genes (*Cd14*, *Tlr1*, *Tlr2* and *Tlr4*) and *Myd88*, as well as inflammatory genes (*Tnf-α*, *Il-1β*, *Ccl-2* and *Il-10*) with quantitative RT-PCR. *t* test, n = 3 - 6 per group, *p* > 0.05 for all tested genes.


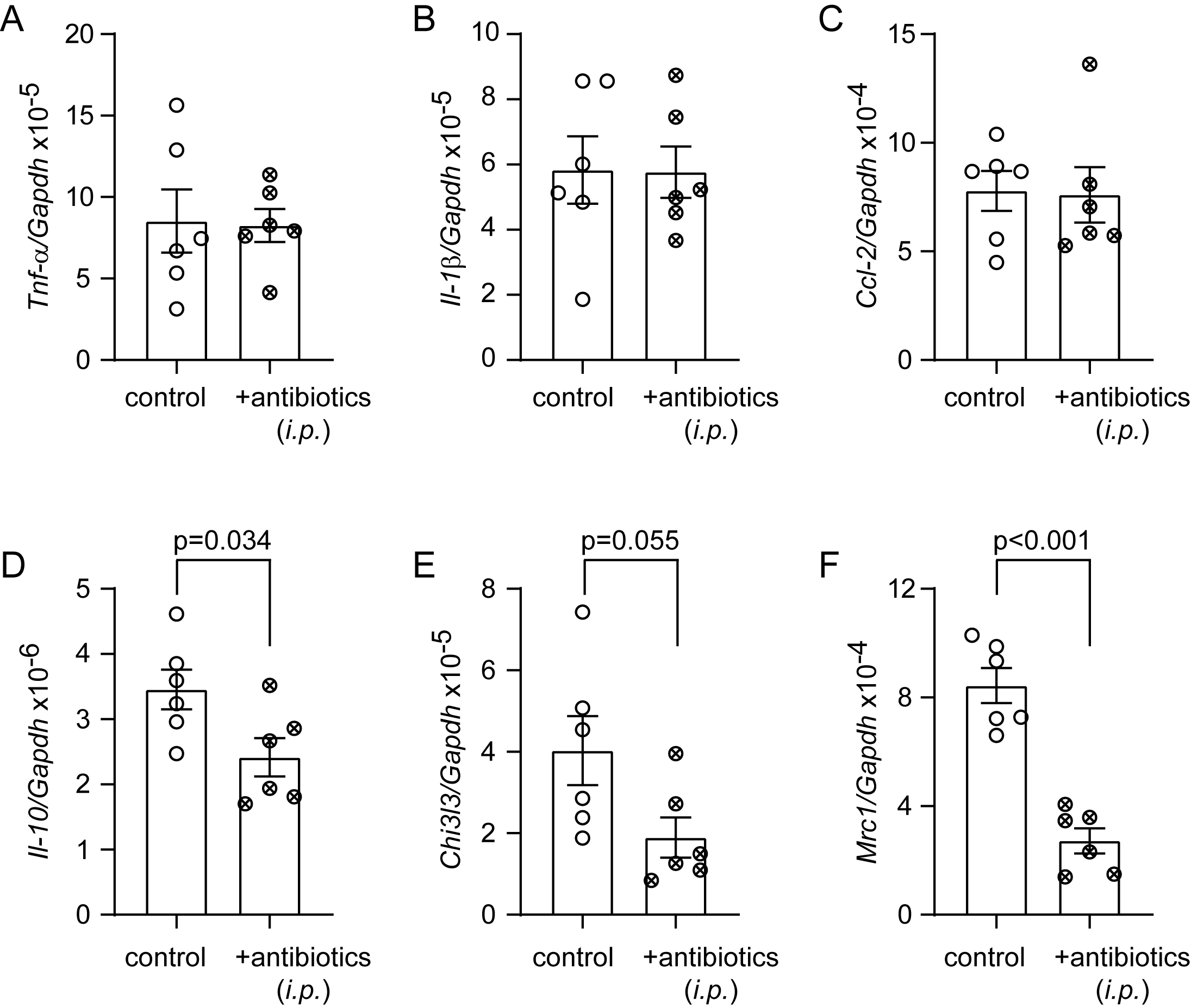


**Supplementary Figure 2, Intraperitoneal injection of an antibiotic cocktail inhibits the transcription of anti-inflammatory but not proinflammatory genes in the brain of APP-transgenic mice.**

Five-month-old APP-transgenic mice were injected (*i.p.*) daily with an antibiotic cocktail (vancomycin, ampicillin, neomycin sulfate, streptomycin, and metronidazole; all from Sigma) for 7 days. Brain tissues were then homogenized in Trizol and total RNA was isolated. Transcripts of inflammatory genes (*Tnf-α*, *Il-1β*, *Ccl-2*, *Il-10*, *Chi3l3* and *Mrc1*) were measured with quantitative RT-PCR. Intraperitoneal injection of antibiotics does not alter the transcription of proinflammatory genes *Tnf-α*, *Il-1β*, and *Ccl-2* (A - C), but decreases the transcripts of antiinflammatory genes *Il-10*, *Chi3l3* and *Mrc1* (D - F). *t* test, n = 6 per group.
